# Stress and anxiety negatively associated with relationship satisfaction and family functioning among low-income overweight or obese pregnant and postpartum women

**DOI:** 10.1371/journal.pmen.0000615

**Published:** 2026-05-19

**Authors:** Mei-Wei Chang, Nathan P. Helsabeck, Holly J. Jones

**Affiliations:** The Ohio State University College of Nursing, Columbus, Ohio, United States of America; PLOS: Public Library of Science, UNITED KINGDOM OF GREAT BRITAIN AND NORTHERN IRELAND

## Abstract

The study explored whether relationship satisfaction, general family functioning, or communication was associated with stress or anxiety. Participants were low-income overweight or obese pregnant (n = 342) or postpartum (n = 365) women. They completed validated surveys measuring relationship satisfaction, general family functioning, and communication as well as stress and anxiety. Regression analysis to test the objective was performed. Separate analyses were conducted for pregnant and postpartum women. Relationship satisfaction (pregnant, β = -0.40, p < .001; postpartum, β = -0.53, p < .001) and general family functioning (pregnant, β = -0.36, p < .001; postpartum, β = -0.51, p < .001) were negatively associated with stress. Similarity, relationship satisfaction (pregnant, β = -0.24, p < .001; postpartum, β = -0.26, p < .001) and general family functioning (pregnant, β = -0.28, p < .001; postpartum, β = -0.23, p < 0.001) were negatively associated with anxiety. However, communication was not associated with stress or anxiety for either pregnant or postpartum women. Among low-income overweight or obese pregnant and postpartum women, those experienced poor relationship satisfaction and general family functioning tended to report higher levels of stress and anxiety.

## Introduction

Social determinants of health (SDOH) are fundamental drivers of health disparities, contributing to disproportionate burden of chronic conditions such as obesity and cardiovascular disease [1]. Within the SDOH framework, income is a key domain associated with psychological distress, such as stress and anxiety [2]. Living in low-income environment increases the risk for psychological distress as well as maternal mortality and morbidity [3]. For women, the prenatal and postpartum periods represent uniquely vulnerable and high-stress life stages. When these transitions intersect with double burden of low-income and overweight or obesity, the risk for adverse health outcomes can be exacerbated [4,5]. Specifically, prenatal psychological distress is associated with hypertensive disorders of pregnancy and premature birth [6]. The impact of this distress extends far beyond pregnancy, it is a predictor of postpartum depression [7], the leading cause of maternal mortality [8]. Low-income postpartum women may experience additional life stressors, for example, care of the newborn and/or family dynamics, thus further contributing to psychological distress [4]. Postpartum psychological distress also has a negative influence on breastfeeding adherence and offspring’s growth, language and cognitive development, gross and fine motor movement, and sleep [9,10]. Therefore, identifying modifiable risk factors for psychological distress is essential to improve maternal health outcomes among pregnant and postpartum women with double burden.

A potential critical, yet under-examined, factor in mitigating psychological distress is the quality of family dynamics. These dynamics include relationship satisfaction, general family functioning, and communication and are negatively influenced by low-income [11–13]. Relationship satisfaction is a vital element of family dynamics [14]. The prenatal and postpartum periods can exert significant pressure on relationship satisfaction, particularly for low-income women who are already navigating multiple, competing life stressors [15–17]. Better relationship satisfaction promotes psychological well-being for women throughout the perinatal period, which extends to the child [18] and serve as a protective factor during stressful events [19,20].

Another critical element of family dynamic is general family functioning defined as adaptability, cohesion, conflict resolution, and affective expression [14]. The transition to parenthood requires substantial shifts in roles and interpersonal relationships, which can further strain general family functioning [21]. While poor general family functioning is associated with psychological distress [22,23], better general family functioning supports the social and emotional skills to cope with stress [19,20].

Communication is an essential component of family dynamics [14] and includes oral speech, nonverbal cues, and affective expressions. Poor communication during prenatal period increases risk for problematic interpersonal patterns during the postpartum period [16] and is associated with psychological distress [24,25].

In summary, relationship satisfaction, general family functioning, and communication are linked to psychological distress. However, prior research has not investigated these concepts within the context of the double burden of low-income and overweight or obesity. Therefore, this study explored whether relationship satisfaction, general family functioning or communication was associated with stress or anxiety in pregnant or postpartum women experiencing double burden. We hypothesized that pregnant or postpartum women with poor relationship satisfaction, general family functioning, or communication would report higher levels of stress and anxiety.

## Methods

### Participants, settings, and procedure

Setting, participants, and procedures of the cross-sectional study has been published [26]. Briefly, participants were recruited between 06/01/2017 – 10/30/2017 through in person invitation while waiting for appointments. They were recruited from a university hospital affiliated Ob/Gyn clinic that serves majority of low-income patients. We also recruited women from the Special Supplemental Nutrition Program for Women, Infants, and Children (WIC) in Ohio. WIC is one of the largest federally funded nutrition programs in U.S. for low-income individuals. Qualified participants must be pregnant or within 1-year postpartum, be at least 18 years old, have body mass index (BMI) at least 25.0 kg/m^2^ computed using self-reported height and weight (pre-pregnancy weight for pregnant women and current weight for postpartum women), and received government assistant programs, for example, WIC, Medicaid.

Ethnic statement. The study procedure was approved by The Ohio State University Institutional Review Boards (IRB #2017B0051). Also, this study was conducted according to the principles expressed in the Declaration of Helsinki. Qualified participants provided a written consent for participation followed by completing a pencil and paper survey.

### Measures

#### Stress.

The short Stress Overload Scale (10 items) was used to measure perception of stress, hereafter stress. The survey has established good validity and reliability [27]. Following current methodological recommendations, we report McDonald’s Omega (ω) as an estimate of internal consistency [28–30]. In the current samples, we found evidence of strong internal consistency for pregnant (ω = 0.929) and postpartum (ω = 0.947) women. Responses range from 1 (not at all) to 5 (a lot). Participants were asked in the past 7 days have your felt (for example) “swamped by your responsibilities?” We summed the 10-item scores to create a composite stress score ranging from 10 to 50. Higher scores mean higher levels of stress.

#### Anxiety.

Generalized Anxiety Disorder Scale (7 items) was used to measure anxiety. This survey has established good validity and reliability [31]. In the current samples, we identified strong internal consistency, ω was 0.916 for pregnant women and 0.933 for postpartum women. Responses range from 0 (not at all) to 3 (nearly every day). Participants were asked in the past 2 weeks, “how often you have been bothered by, for example, worrying too much about different things.” We summed the 7- item scores to create a composite anxiety score ranging from 0 to 21. Higher scores mean higher levels of anxiety.

### Relationship satisfaction

Relationship Assessment Scale (7 items) was used to measure relationship satisfaction with partners [32]. This survey has established good validity and reliability [33]. In the current samples internal consistency was strong, ω was 0.896 for pregnant women and 0.906 for postpartum women. Responses range from 1 (low satisfaction) to 5 (high satisfaction). Participants were asked, for example, “how good is your relationship compared to most?” We summed the 7-item scores to create a composite relationship satisfaction score ranging from 7 to 35. Higher scores mean better relationship satisfaction.

### General family functioning

A subscale of the McMaster Family Assessment Device (FAD) was used to assess general family functioning (12 items). The FAD has established good validity and reliability [34]. In the current samples internal consistency was strong, ω was 0.859 for pregnant women and 0.852 for postpartum women. Responses range from 1 (strongly disagree) to 4 (strongly agree). Participants were asked questions related to general family functioning: quality of communication, adaptability (adjusting to changing stressors), cohesion (emotional connection between family members), conflict resolution (healthy strategies to resolve conflict), and affective expression (openness to share emotion). For example, “in times of crisis we can turn to each other for support.” We summed 12-item scores to create a composite general family functioning score ranging from 12 to 48. Higher scores indicate better general family functioning.

### Communication

A subscale of the FAD was used to assess communication (6 items) [34]. In the current samples internal consistency was moderate, ω was 0.636 for pregnant women and 0.610 for postpartum women. Responses range from 1 (strongly disagree) to 4 (strongly agree). The communication scale focuses on assessing clarity and directness of information exchange (message directly to the intended person) among family members. For example, “when someone is upset, the others know why.” We summed 6-item of communication score to create a composite communication score ranging from 6 to 24. Higher scores indicate better communication.

### Statistical analysis

We used descriptive statistics and frequencies to describe the sample demographics (N = 707, pregnant = 342, postpartum 365). The independent variables of interest were relationship satisfaction, general family functioning, and communication. The dependent variables were stress and anxiety. Covariates included race, education, and gestational age in weeks or postpartum in weeks of the youngest child. These covariates were selected because they influence stress and anxiety. Participants (11% pregnant women and 19% postpartum women) who were not in relationship were asked to skip relationship satisfaction questions. A small portion of participants (2% for pregnant and 3% for postpartum) had missing data, because their ride arrived prior to completing the survey. Performing multiple imputation is not recommended when missingness is < 5% [35,36]. Nevertheless, we conducted a series of sensitivity tests that included all independent and dependent variables as well as covariates to determine if analytic sample (participants without missing data) differed from the full sample (participants who responded to the survey with or without missing data). Results of our sensitivity analysis showed no group difference between the full and the analytic samples on any variable described above for either pregnant or postpartum women. Therefore, we are confident that missing at random is held, and preforming complete case analysis is appropriate [35,36].

To determine the association between independent and dependent variables, we conducted a series of regression models. Prior to conducting the analysis, we checked all variables to assure that they met the assumptions for linear regression. We found that all variables had normally distributed residuals and consistent residual variability, as well as demonstrated a linear relationship between independent and dependent variables. Additionally, we did not detect outliers in our data. However, we did detect moderate correlations between our independent variables (pregnant women: r = .323 to .502, post-partum women r = .238 to.520). To avoid potential multicollinearity, we, therefore, conducted single regression model (1 dependent and 1 independent variables) while controlling for covariates (described above). This approach has the added value of allowing us to examine if there were differences in which covariates were significantly related to each independent variable. All analyses were conducted separately for pregnant and postpartum women. A two-tailed alpha of .05 was set to determine statistical significance and 95% confidence intervals are reported for parameter estimates. The proportion of variability explained by the model is reported using R^2^ and adjusted R^2^ values. We used R 4.2.2 to conduct all statistical analysis [37].

## Results

### Demographics

Table 1 summarizes demographics of pregnant and postpartum women. The pregnant and postpartum samples had a similar mean age and BMI. Both groups represented a racially diverse sample. Furthermore, the distribution of education, smoking and employment status were comparable across both groups.

**Table 1:** Demographic description of the sample.

	Total	Pregnant	Postpartum
	(N = 707)	(N = 342)	(N = 365)
**BMI**			
Mean (SD)	34.8 (7.28)	35.8 (7.70)	34.0 (6.75)
Median [Min, Max]	33.3 [24.0, 80.6]	34.5 [24.2, 80.6]	32.3 [24.0, 65.7]
**Mother’s Age (Years)**			
Mean (SD)	27.4 (5.52)	27.5 (5.69)	27.3 (5.37)
Median [Min, Max]	27.0 [18.0, 45.0]	27.0 [18.0, 45.0]	27.0 [18.0, 43.0]
**Gestational Age (Weeks)**			
Mean (SD)	24.3 (9.76)	24.3 (9.76)	N/A
Median [Min, Max]	25.0 [1.00, 40.0]	25.0 [1.00, 40.0]	N/A
**Age of youngest child (Weeks)**			
Mean (SD)	17.9 (14.3)	N/A	17.9 (14.3)
Median [Min, Max]	16.0 [0, 48.0]	N/A	16.0 [0, 48.0]
**Race**			
Missing	14 (2.0%)	5 (1.5%)	9 (2.5%)
Black	357 (50.5%)	194 (56.7%)	163 (44.7%)
White	323 (45.7%)	137 (40.1%)	186 (51.0%)
Asian	2 (0.3%)	1 (0.3%)	1 (0.3%)
Pacific Islander	7 (1.0%)	3 (0.9%)	4 (1.1%)
Native American	4 (0.6%)	2 (0.6%)	2 (0.5%)
**Educational**			
Less than High School	81 (11.5%)	54 (15.8%)	27 (7.4%)
High School	274 (38.8%)	134 (39.2%)	140 (38.4%)
Some College	259 (36.6%)	118 (34.5%)	141 (38.6%)
College Graduate	93 (13.2%)	36 (10.5%)	57 (15.6%)
**Smoking**			
Never smoked	330 (46.7%)	147 (43.0%)	183 (50.1%)
Smoked, but quit	249 (35.2%)	133 (38.9%)	116 (31.8%)
Smoker	128 (18.1%)	62 (18.1%)	66 (18.1%)
**Employment**			
Full time	249 (35.2%)	117 (34.2%)	132 (36.2%)
Part time	104 (14.7%)	51 (14.9%)	53 (14.5%)
Unemployed	217 (30.7%)	113 (33.0%)	104 (28.5%)
Homemaker	87 (12.3%)	39 (11.4%)	48 (13.2%)
Self-employed	18 (2.5%)	8 (2.3%)	10 (2.7%)
Student	11 (1.6%)	3 (0.9%)	8 (2.2%)
Other	21 (3.0%)	11 (3.2%)	10 (2.7%)

### The stress and anxiety associations

Table 2 presents means and median of variable of interest. Table 3 shows associations between relationship satisfaction, general family functioning, communication and stress for pregnant and postpartum women. Among pregnant women, we observed negative associations between relationship satisfaction and stress (*β* = -0.398, SE = 0.074, *p* < .001) and between general family functioning and stress (*β* = -0.355, SE = 0.089, *p* < .001) but no association between communication and stress after controlling for covariates. The relationship satisfaction and general family functioning models explained 12.0% and 8.0% of variability in stress, respectively. Similar results were observed in the postpartum women when controlling for covariates. There were negative associations between relationship satisfaction and stress (*β* = -0.527, SE = 0.084, *p* < .001) and between general family functioning and stress (*β* = -0.51, SE = 0.1, *p* < .001). Again, communication was not associated with stress. While the relationship satisfaction model explained 19% of variability in stress, the general family functioning model explained 13% of variability in stress.

**Table 2:** Descriptive Statistics of Variables of Interest.

	Total	Pregnant	Postpartum
	(N = 707)	(n = 342)	(n = 365)
**Stress**			
Mean (SD)	22.6 (10.1)	22.5 (9.62)	22.7 (10.5)
Median [Min, Max]	21.0 [10.0, 50.0]	22.0 [10.0, 50.0]	21.0 [10.0, 50.0]
**Anxiety**			
Mean (SD)	7.14 (5.85)	7.78 (5.77)	6.53 (5.87)
Median [Min, Max]	6.00 [0, 21.0]	7.00 [0, 21.0]	5.00 [0, 21.0]
**Relationship Satisfaction** ^**1**^			
Mean (SD)	28.1 (6.78)	27.6 (6.90)	28.7 (6.62)
Median [Min, Max]	30.0 [4.00, 35.0]	30.0 [7.00, 35.0]	31.0 [4.00, 35.0]
**General Family Functioning** ^**2**^			
Mean (SD)	37.6 (5.55)	36.9 (5.70)	38.2 (5.32)
Median [Min, Max]	38.0 [15.0, 46.0]	37.0 [15.0, 46.0]	39.0 [20.0, 46.0]
**Communication** ^**3**^			
Mean (SD)	16.2 (2.71)	16.0 (2.80)	16.3 (2.62)
Median [Min, Max]	16.0 [6.00, 24.0]	16.0 [6.00, 24.0]	17.0 [6.00, 24.0]

^1^Total = 600 (pregnant 273, postpartum 297).

^2^Total = 688 (pregnant 334, postpartum 354).

^3^Total = 690 (pregnant 335, postpartum 355).

**Table 3:** Associations with Stress in Pregnant and Postpartum Women*.*

Pregnant	Relationship Satisfaction				General Family Functioning				Communication			
	β	SE	*p*	95% CI	β	SE	*p*	95% CI	β	SE	*p*	95% CI
Intercept	27.181	3.027	<.001	[21.2, 33.1]	27.793	3.813	<.001	[20.3, 35.3]	14.607	3.624	<.001	[7.5, 21.7]
Estimate^1^	-0.398	0.074	<.001	[-0.54, -0.25]	-0.355	0.089	<.001	[-0.53, -0.18]	0.063	0.185	.732	[-0.3, 0.43]
Gestation weeks	0.005	0.052	.930	[-0.1, 0.11]	0.033	0.052	.526	[-0.07, 0.14]	0.025	0.053	.636	[-0.08, 0.13]
Race	-0.052	0.044	.245	[-0.14, 0.04]	-0.039	0.046	.403	[-0.13, 0.05]	-0.039	0.047	.411	[-0.13, 0.05]
Education	1.710	0.564	.003	[0.6, 2.82]	2.076	0.568	<.001	[0.96, 3.19]	1.859	0.576	.001	[0.73, 2.99]
Observations	303				334				335			
R2	0.120				0.079				0.035			
Adjusted R2	0.108				0.068				0.024			
F Statistic	10.15 (df = 4; 298); p <.001				7.028 (df = 4; 329); p <.001				3.011 (df = 4; 330); p =.018			
**Postpartum**	**Relationship Satisfaction**				**General Family Functioning**				**Communication**			
	**β**	**SE**	***p***	**95% CI**	**β**	**SE**	***p***	**95% CI**	**β**	**SE**	***p***	**95% CI**
Intercept	31.488	3.493	<.001	[24.6, 38.4]	35.319	4.39	<.001	[26.7, 44]	21.206	4.097	<.001	[13.2, 29.3]
Estimate^1^	-0.527	0.084	<.001	[-0.69, -0.36]	-0.510	0.100	<.001	[-0.71, -0.31]	-0.298	0.206	.150	[-0.7, 0.11]
Postpartum Weeks	0.155	0.039	<.001	[0.08, 0.23]	0.154	0.037	<.001	[0.08, 0.23]	0.183	0.038	<.001	[0.11, 0.26]
Race	-0.046	0.043	.283	[-0.13, 0.04]	-0.032	0.036	.377	[-0.1, 0.04	-0.031	0.037	.404	[-0.1, 0.04]
Education	0.985	0.638	.123	[-0.27, 2.24]	1.128	0.617	.068	[-0.09, 2.34]	0.832	0.632	.189	[-0.41, .07]
Observations	297				354				355			
R2	0.185				0.134				0.075			
Adjusted R2	0.174				0.124				0.064			
F Statistic	16.56 (df = 4; 292); p <.001				13.48 (df = 4; 349); p <.001				7.077 (df = 4; 350); p <.001			

^1^Model estimates for the independent variable (relationship satisfaction, general family function, or communication) in each model. β = beta coefficient. SE = standard error of the estimate. CI = confidence interval. R^2^ = coefficient of determination of the estimate. CI = confidence interval.

Table 4 shows associations between relationship satisfaction, general family functioning, communication and anxiety for pregnant and postpartum women. For pregnant women, we found negative associations between relationship satisfaction and anxiety (*β* = -0.236, SE = 0.044, *p* < .001) and between general family functioning and anxiety (*β* = -0.277, SE = 0.054, *p* < .001) after controlling for covariates. However, communication was not associated with anxiety. The relationship satisfaction model explained 10.0% of variability in anxiety, and the general family functioning model explained 8.0% variability in anxiety. We observed the same pattern of anxiety associations for postpartum women. After controlling for covariates, our results showed negative associations between relationship satisfaction and anxiety (*β* = -0.260, SE = 0.049, *p* < .001) and between general family functioning and anxiety (*β* = -0.232, SE = 0.058, *p* < .001). Again, communication was not associated with anxiety. The relationship satisfaction model explained 11% and the general family functioning model explained 6.1% variability in anxiety.

**Table 4:** Associations with Anxiety in Pregnant and Postpartum Women.

Pregnant	Relationship Satisfaction				General Family Functioning				Communication			
	β	SE	*p*	95% CI	β	SE	*p*	95% CI	β	SE	*p*	95% CI
Intercept	14.314	1.824	<.001	[10.7, 17.9]	16.831	2.299	<.001	[12.4, 21.4]	7.828	2.220	<.001	[3.5, 12.2]
Estimate^1^	-0.236	0.044	<.001	[-0.32, -0.15]	-0.277	0.054	<.001	[-0.38, -0.17]	-0.038	0.113	.741	[-0.26, 0.19]
Gestation weeks	-0.054	0.031	.087	[-0.12, 0.01]	-0.021	0.031	.501	[-0.08, 0.04]	-0.026	0.033	.421	[-0.09, 0.04]
Race	-0.016	0.027	.561	[-0.07, 0.04]	-0.006	0.028	.832	[-0.06, 0.05]	-0.007	0.029	.817	[-0.06, 0.05]
Education	0.322	0.340	.344	[-0.35, 0.99]	0.505	0.343	.142	[-0.17, 1.18]	0.367	0.353	.299	[-0.33, 1.06]
Observations	303				334				335			
R2	0.101				0.079				0.005			
Adjusted R2	0.089				0.068				-0.007			
F Statistic	8.374 (df = 4, 298); p <.001				7.041 (df = 4; 329); p <.001				0.4396 (df = 4; 330); p = .78			
**Postpartum**	**Relationship Satisfaction**				**General Family Functioning**				**Communication**			
	**β**	**SE**	***p***	**95% CI**	**β**	**SE**	***p***	**95% CI**	**β**	**SE**	***p***	**95% CI**
Intercept	12.126	2.067	<.001	[8.1, 16.2]	13.662	2.561	<.001	[8.6, 18.7]	7.657	2.359	0.001	[3.02, 12.3]
Estimate^1^	-0.260	0.049	<.001	[-0.36, -0.16]	-0.232	0.058	<.001	[-0.35, -0.12]	-0.162	0.119	.173	[-0.4, 0.07]
Postpartum weeks	0.041	0.023	.079	[-0.01, 0.09]	0.041	0.022	.063	[-0.01, 0.08]	0.054	0.022	.015	[0.01, 0.1]
Race	-0.023	0.025	.354	[-0.07, 0.03]	-0.008	0.021	.717	[-0.05, 0.03]	-0.007	0.021	.745	[-0.05, 0.04]
Education	0.336	0.377	.388	[-0.42, 1.07]	0.286	0.36	.428	[-0.42, 1]	0.152	0.364	.675	[-0.56, 0.87]
Observations	297				354				355			
R2	0.108				0.061				0.023			
Adjusted R2	0.096				0.05				0.012			
F Statistic	8.839 (df = 4; 292); p <.001				5.621 (df = 4; 349); p <.001				2.072 (df = 4; 350); p = .084			

^1^Model estimates for the independent variable (relationship satisfaction, general family function, or communication) in each model. β = beta coefficient. SE = standard error of the estimate. CI = confidence interval. R^2^ = coefficient of determination of the estimate. CI = confidence interval.

## Discussion

The study explored whether relationship satisfaction, general family functioning, or communication was associated with psychological distress among pregnant and postpartum women facing double burden of low-income and overweight or obesity. Our findings partially supported the initial hypotheses. Pregnant and postpartum women who reported poor relationship satisfaction or family functioning were likely to report higher levels of stress and anxiety. However, the association between communication and psychological distress yielded null results, contradicting our hypothesis.

To assess the practical significance of these associations, we applied the Minimal Clinically Important Difference (MCID) using a distribution-based approach [38]. We defined the MCID as 0.5 standard deviation of stress and anxiety measures [38]. Therefore, a clinically meaningful reduction in stress would be a 4.8-point for pregnant women and a 5.4-point for postpartum women. Based on our observed regression coefficients (pregnant: β = 0.40; postpartum: β = 0.53), a 1-point reduction in stress requires a 2.5-point (pregnant) or approximately 2.0-point (postpartum) improvement in relationship satisfaction. Achieving the MCID would,therefore, require a 12-point (pregnant) and a 10-point (postpartum) increase in relationship satisfaction. Given the 7–35 scale range of the relationship satisfaction survey, these increments present approximately one-third of the total possible score. Nevertheless, future prospective studies of this population are necessary to verify these findings prior to implementing targeted interventions to enhance relationship satisfaction.

The negative association between relationship satisfaction and stress observed in this study is consistent with prospective study of women [39]. Similarly, our findings regarding anxiety align with prior research [40–43]. Notably, the association between relationship satisfaction and psychological distress could be bidirectional [43]. Poor relationship satisfaction may exacerbate stress and anxiety, and higher levels of psychological distress can, in turn, negatively influence women’s perception of relationships with a partner.

Our findings indicate that a 3-point improvement in general family functioning is required to achieve a 1-point reduction in stress during pregnancy. Similarly, an improvement of approximately 2.0 points in general family functioning is necessary to reach the same reduction in the postpartum period. Achieving the MCID would, therefore, require a substantial enhancement in general family functioning, representing a 25% (pregnant) and 21% (postpartum) improvement based on a total possible score of 48. We observed a moderate correlation between general family functioning and relationship satisfaction (r = .502 pregnant and .520 for postpartum), suggesting a bidirectional association. In this dynamic, elevated stress or anxiety may compromise both constructs. Conversely, poor family functioning and low relationship satisfaction may amplify psychological distress. Consequently, prospective studies are warranted to investigate the longitudinal interrelationships among these variables.

Our results show that poor general family functioning was associated with higher levels of stress and anxiety in pregnant and postpartum women. Our findings diverge from a prior study of pregnant women in China, which reported no association between general family functioning and stress [24]. Distinct cultural differences between American and Chinese pregnant women may account for these conflicting results Nevertheless, our findings are in line with prior studies demonstrating that a negative association between family functioning and stress in African American adults [22] and young adults [44]. Similarly, our results are consistent with research linking poor family functioning and anxiety in adolescent [23,45,46].

Our non-significant findings between communication and psychological distress may reflect the specific components of communication assessed in this study. While daily interactions involve a complex array of verbal and non-verbal cues, our assessment was limited to the clarity and directness of communication. Nevertheless, our null findings are inconsistent with prior research. For instance, a study of Chinese pregnant women with hypertensive disorders found that a lack of clear and direct communication was positively associated with higher levels of stress [24]. Similarly, previous research has shown that poor parent-child communication exacerbated anxiety among adolescents [25]. Furthermore, better affective communication has been associated with decreased stress in pediatric setting [47] and improve stress recovery in college students [48]. Other research suggests that while communication focused on hope evocation was associated with lower levels of anxiety, ruminative messages characterized by the repetitive discussion of negative feelings were associated with higher levels of anxiety [49]. Future research particularly prospective studies involving low-income overweight or obese pregnant and postpartum women should utilize multidimensional assessments. Such approaches are necessary to identify which specific communication styles are most saliently linked to psychological distress.

### Limitations

Several limitations of this study should be noted. First, the assessment of communication was restricted to clarity and directness of verbal interactions. This focus excludes other significant channels, such as nonverbal cues, digital communication (e.g., text messaging or email) and specific affective components. The reliance on self-reported height and weight to calculate BMI may have introduced social desirability or recall bias, potentially compromising the accuracy of the anthropometric data. Also, anxiety was self-reported rather than diagnosis. Participants were recruited from a specific geographic location. We did not collect data on prior history of mental health or immediate environmental stressors (e.g., housing instability). As with any study there may be residual confounding related to important contributions of unmeasured variables that are not included in the models. Furthermore, we conducted single rather than multiple regression. Consequently, we are unable to detect the independent effects of relationship satisfaction, general family functioning and communication. Finally, our sample included low-income overweight or obese women across a broad range of gestational age and postpartum duration. Collectively, these factors limit the generalizability of our findings.

## Conclusions

Our findings showed that poor relationship satisfaction and general family functioning were significantly associated with higher levels of psychological distress among pregnant and postpartum women with double burden. The lack of association between communication and psychological distress suggests that assessing oral interaction in isolation may not be sufficient. Therefore, future research should assess different channels of communication, including nonverbal cues and digital interactions, to more accurately reflect the complexity of familial dynamics. Prospective cohort studies are warranted to validate our findings and elucidate the bidirectional associations and temporal dynamics of these relationships across the prenatal and postpartum periods.

## Supporting information

S1 File Pregnant women code book. (DOCX)

S2 File Postpartum women code book. (DOCX)

S1 Data Pregnant women data. (XLSX)

S2 Data Postpartum women data. (XLSX)

## References

[1] Powell-Wiley TM, Baumer Y, Baah FO, Baez AS, Farmer N, Mahlobo CT, et al. Social Determinants of Cardiovascular Disease. Circ Res. 2022;130(5):782–99. doi: 10.1161/CIRCRESAHA.121.319811 35239404 PMC8893132

[2] Silva M, Loureiro A, Cardoso G. Social determinants of mental health: a review of the evidence. The European Journal of Psychiatry. 2016;30(4):259–92.

[3] Wang E, Glazer KB, Howell EA, Janevic TM. Social Determinants of Pregnancy-Related Mortality and Morbidity in the United States: A Systematic Review. Obstet Gynecol. 2020;135(4):896–915. doi: 10.1097/AOG.0000000000003762 32168209 PMC7104722

[4] Mollard E, Kupzyk K, Moore T. Postpartum stress and protective factors in women who gave birth in the United States during the COVID-19 pandemic. Womens Health (Lond). 2021;17:17455065211042190. doi: 10.1177/17455065211042190 34465268 PMC8414615

[5] Yang L, Zhao Y, Wang Y, Liu L, Zhang X, Li B, et al. The Effects of Psychological Stress on Depression. Curr Neuropharmacol. 2015;13(4):494–504. doi: 10.2174/1570159x1304150831150507 26412069 PMC4790405

[6] Preis H, Mahaffey B, Pati S, Heiselman C, Lobel M. Adverse Perinatal Outcomes Predicted by Prenatal Maternal Stress Among U.S. Women at the COVID-19 Pandemic Onset. Ann Behav Med. 2021;55(3):179–91. doi: 10.1093/abm/kaab005 33724334 PMC7980766

[7] Obrochta CA, Chambers C, Bandoli G. Psychological distress in pregnancy and postpartum. Women Birth. 2020;33(6):583–91. doi: 10.1016/j.wombi.2020.01.009 32035798

[8] Trost SL, Beauregard JL, Smoots AN, Ko JY, Haight SC, Moore Simas TA, et al. Preventing Pregnancy-Related Mental Health Deaths: Insights From 14 US Maternal Mortality Review Committees, 2008-17. Health Affairs. 2021;40(10):1551–9. 34606354 10.1377/hlthaff.2021.00615 PMC11135281

[9] Gila-Díaz A, Carrillo GH, López de Pablo ÁL, Arribas SM, Ramiro-Cortijo D. Association between Maternal Postpartum Depression, Stress, Optimism, and Breastfeeding Pattern in the First Six Months. Int J Environ Res Public Health. 2020;17(19):7153. doi: 10.3390/ijerph17197153 33007816 PMC7579508

[10] Oyetunji A, Chandra P. Postpartum stress and infant outcome: A review of current literature. Psychiatry Res. 2020;284:112769. doi: 10.1016/j.psychres.2020.112769 31962260

[11] Mansfield AK, Dealy JA, Keitner GI. Family functioning and income: does low-income status impact family functioning?. The Family Journal. 2013;21(3):297–305.

[12] Karney BR. Socioeconomic Status and Intimate Relationships. Annual Review of Psychology. 2021;72(1):391–414. 10.1146/annurev-psych-051920-013658 PMC8179854 32886585

[13] Banovcinova A, Levicka K. The Impact of the Financial Income on the Family Communication. rrem. 2015;7(2):35–46. doi: 10.18662/rrem/2015.0702.03

[14] Lewandowski AS, Palermo TM, Stinson J, Handley S, Chambers CT. Systematic review of family functioning in families of children and adolescents with chronic pain. J Pain. 2010;11(11):1027–38. doi: 10.1016/j.jpain.2010.04.005 21055709 PMC2993004

[15] Doss BD, Rhoades GK. The transition to parenthood: impact on couples’ romantic relationships. Curr Opin Psychol. 2017;13:25–8. doi: 10.1016/j.copsyc.2016.04.003 28813289

[16] Doss BD, Rhoades GK, Stanley SM, Markman HJ. The effect of the transition to parenthood on relationship quality: an 8-year prospective study. J Pers Soc Psychol. 2009;96(3):601–19. doi: 10.1037/a0013969 19254107 PMC2702669

[17] Randall AK, Bodenmann G. Stress and its associations with relationship satisfaction. Curr Opin Psychol. 2017;13:96–106. doi: 10.1016/j.copsyc.2016.05.010 28813303

[18] Hoffmann L, Hilger N, Riolino E, Lenz A, Banse R. Partner support and relationship quality as potential resources for childbirth and the transition to parenthood. BMC Pregnancy Childbirth. 2023;23(1):435. doi: 10.1186/s12884-023-05748-6 37312055 PMC10261844

[19] Gouveia F, Gouveia-Pereira M, Portugal A. COVID-19, family and love relationships: perception of stress, relational quality, family functioning and online infidelity behaviors. Journal of Couple & Relationship Therapy. 2024;23(3):213–28.

[20] Hooper A, Hustedt JT, Slicker G, Hallam RA, Gaviria-Loaiza J. Linking early head start children’s social-emotional functioning with profiles of family functioning and stress. J Fam Psychol. 2023;37(1):153–60. doi: 10.1037/fam0001014 35925719

[21] Martins CA. Transition to parenthood: consequences on health and well-being. A qualitative study. Enferm Clin (Engl Ed). 2019;29(4):225–33. doi: 10.1016/j.enfcli.2018.04.005 29914790

[22] Pollock ED, Kazman JB, Deuster P. Family functioning and stress in African American families: A strength-based approach. Journal of Black Psychology. 2015;41(2):144–69.

[23] Farmakopoulou I, Lekka M, Gkintoni E. Clinical Symptomatology of Anxiety and Family Function in Adolescents-The Self-Esteem Mediator. Children (Basel). 2024;11(3):338. doi: 10.3390/children11030338 38539373 PMC10969624

[24] Xing S, Wan L, Fu A, Liu W, Lin L, Wang C, et al. Correlation analysis of stress and family function and coping modes in pregnant women with pregnancy-induced hypertension syndrome. Ann Palliat Med. 2021;10(11):11688–94. doi: 10.21037/apm-21-2662 34872293

[25] Romero-Abrio A, Martínez-Ferrer B, Musitu-Ferrer D, León-Moreno C, Villarreal-González ME, Callejas-Jerónimo JE. Family Communication Problems, Psychosocial Adjustment and Cyberbullying. Int J Environ Res Public Health. 2019;16(13):2417. doi: 10.3390/ijerph16132417 31288393 PMC6651853

[26] M-W C, DT W, J P, RE L. Associations between Stress, Body Mass Index, Demographics and Eating Behaviors in Low-Income Overweight or Obese Women. AustinJPublicHealthEpidemiol. 2022;9(1). doi: 10.26420/austinjpublichealthepidemiol.2022.1118

[27] Amirkhan JH. A Brief Stress Diagnostic Tool: The Short Stress Overload Scale. Assessment. 2018;25(8):1001–13. doi: 10.1177/1073191116673173 30392415

[28] Cronbach LJ, Shavelson RJ. My Current Thoughts on Coefficient Alpha and Successor Procedures. Educational and Psychological Measurement. 2004;64(3):391–418. doi: 10.1177/0013164404266386

[29] Flora DB. Your Coefficient Alpha Is Probably Wrong, but Which Coefficient Omega Is Right? A Tutorial on Using R to Obtain Better Reliability Estimates. Advances in Methods and Practices in Psychological Science. 2020;3(4):484–501. doi: 10.1177/2515245920951747

[30] McNeish D. Thanks coefficient alpha, we’ll take it from here. Psychol Methods. 2018;23(3):412–33. doi: 10.1037/met0000144 28557467

[31] Spitzer RL, Kroenke K, Williams JBW, Löwe B. A brief measure for assessing generalized anxiety disorder: the GAD-7. Arch Intern Med. 2006;166(10):1092–7. doi: 10.1001/archinte.166.10.1092 16717171

[32] Hendrick SS. A Generic Measure of Relationship Satisfaction. Journal of Marriage and the Family. 1988;50(1):93. doi: 10.2307/352430

[33] Vaughn MJ, Matyastik Baier ME. Reliability and validity of the relationship assessment scale. The American Journal of Family Therapy. 1999;27(2):137–47. doi: 10.1080/019261899262023

[34] Epstein NB, Baldwin LM, Bishop DS. The McMaster family assessment device*. J Marital Family Therapy. 1983;9(2):171–80. doi: 10.1111/j.1752-0606.1983.tb01497.x

[35] Dettori JR, Norvell DC, Chapman JR. The Sin of Missing Data: Is All Forgiven by Way of Imputation?. Global Spine J. 2018;8(8):892–4. doi: 10.1177/2192568218811922 30560043 PMC6293424

[36] Jakobsen JC, Gluud C, Wetterslev J, Winkel P. When and how should multiple imputation be used for handling missing data in randomised clinical trials - a practical guide with flowcharts. BMC Med Res Methodol. 2017;17(1):162. doi: 10.1186/s12874-017-0442-1 29207961 PMC5717805

[37] R: A language and environment for statistical computing. https://www.R-project.org/

[38] Norman GR, Sloan JA, Wyrwich KW. Interpretation of changes in health-related quality of life: the remarkable universality of half a standard deviation. Med Care. 2003;41(5):582–92. doi: 10.1097/01.MLR.0000062554.74615.4C 12719681

[39] Pauly T, Lüscher J, Berli C, Scholz U. Dynamic associations between stress and relationship functioning in the wake of COVID-19: Longitudinal data from the German family panel (pairfam). J Soc Pers Relat. 2022;39(11):3183–203. doi: 10.1177/02654075221092360 38603129 PMC9047667

[40] Pieh C, O Rourke T, Budimir S, Probst T. Relationship quality and mental health during COVID-19 lockdown. PLoS One. 2020;15(9):e0238906. doi: 10.1371/journal.pone.0238906 32915878 PMC7485771

[41] Leach LS, Butterworth P, Olesen SC, Mackinnon A. Relationship quality and levels of depression and anxiety in a large population-based survey. Soc Psychiatry Psychiatr Epidemiol. 2013;48(3):417–25. doi: 10.1007/s00127-012-0559-9 22875222

[42] Salehi F, Shahhosseini Z. Association Between Women’s Marital Satisfaction and Anxiety During Pregnancy. Iran J Psychiatry Behav Sci. 2017;11(3). doi: 10.17795/ijpbs-7937

[43] Kasalova P, Prasko J, Holubova M, Vrbova K, Zmeskalova D, Slepecky M, et al. Anxiety disorders and marital satisfaction. Neuro Endocrinol Lett. 2018;38(8):555–64. 29504737

[44] Chapman LK, Woodruff-Borden J. The impact of family functioning on anxiety symptoms in African American and European American young adults. Personality and Individual Differences. 2009;47(6):583–9. doi: 10.1016/j.paid.2009.05.012

[45] Guo Z, Zhao J, Peng J. Family function and anxiety among junior school students during the COVID-19 pandemic: a moderated mediation model. Front Psychiatry. 2023;14:1217709. doi: 10.3389/fpsyt.2023.1217709 37426107 PMC10324413

[46] Hua Y, Jiang W, He Y, Zheng X, Huang C, Guo L, et al. Associations of recent stressful life events with anxiety symptoms among Chinese adolescents with a consideration of family functioning. Eur J Psychotraumatol. 2024;15(1):2337577. doi: 10.1080/20008066.2024.2337577 38597558 PMC11008314

[47] Gemmiti M, Hamed S, Lauber-Biason A, Wildhaber J, Pharisa C, Klumb PL. Pediatricians’ affective communication behavior attenuates parents’ stress response during the medical interview. Patient Educ Couns. 2017;100(3):480–6. doi: 10.1016/j.pec.2016.09.006 27816315

[48] Floyd K, Mikkelson AC, Tafoya MA, Farinelli L, La Valley AG, Judd J, et al. Human affection exchange: XIII. Affectionate communication accelerates neuroendocrine stress recovery. Health Commun. 2007;22(2):123–32. doi: 10.1080/10410230701454015 17668992

[49] Chadwick AE, Zoccola PM, Figueroa WS, Rabideau EM. Communication and Stress: Effects of Hope Evocation and Rumination Messages on Heart Rate, Anxiety, and Emotions After a Stressor. Health Commun. 2016;31(12):1447–59. doi: 10.1080/10410236.2015.1079759 27054822

