# Supplementary material for: Stress and anxiety negatively associated with relationship satisfaction and family functioning among low-income overweight or obese pregnant and postpartum women
Source: PLOS Ment Health. 2026 May 19;3(5):e0000615. doi: 10.1371/journal.pmen.0000615 (PMC13186386; doi:10.1371/journal.pmen.0000615)
Supplement: S2 File — (DOCX) [file pmen.0000615.s002.docx]

**Postpartum women code book**

| Variable | Label |
| --- | --- |
| asurveyID | Survey ID |
| aupregnantwk | How far along you are in your pregnancy (in weeks) |
| auage | What is your age |
| aufirstap | Is this your first pregnancy |
| auhispanic | Would you describe yourself as Hispanic |
| aurace | Race |
| ausmoke | Smoking status |
| auedu | The highest level of education that you have completed |
| auemploy1 | Employment status |
| ausswamped | Swamped by your responsibilities |
| ausnotime | Wasn't enough time to get to everything |
| ausrush | Like you were rushed |
| auspillingup | Like things kept piling up |
| ausheavyload | Like you were carrying a heavy load |
| ausinadequate | Inadequate |
| ausodds | Odds were against you |
| ausnothing | Nothing was going right |
| ausnoescape | There was no escape |
| ausgivingup | Just giving up |
| auanervous | Feeling nervous, anxious, or on edge |
| auastopworry | Not being able to stop or control worrying |
| auaworrymuch | Worrying too much about different things |
| auatrourelax | Trouble relaxing |
| auarestless | Being so restless that it's hard to sit still |
| auaannoyed | Becoming easily annoyed or irritable |
| auaafraid | Feeling afraid as if something awful might happen |
| auadifficult | How difficult have these made it for you to do your work, take care of things at home, or get along with other people |
| audlaugh | Laugh and see the funny side of things |
| audenjoyment | Looked forward with enjoyment to things |
| audblamedme | Blamed myself unnecessarily when things went wrong |
| audanxious | Anxious or worried for no good reason |
| audscared | Felt scared or panicky for no very good reason |
| audontopofme | Things have been getting on top of me |
| audunhappys | So unhappy that I have had difficulty sleeping |
| audsad | I have felt sad or miserable |
| audunhappyc | So unhappy that I have been crying |
| audharmingme | The thought of harming myself has occured to me |
| aubfleshfirm | Have you worried about your flesh not being firm enough |
| aubsmamtfood | Has eating even a small amount of food made you feel fat |
| aubavoidclo | Have you avoided wearing clothes which make you aware of the shape of your body |
| aubashame | Have you felt ashamed of your body |
| aubworryshape | Has worry about your shape made you diet |
| aubstomachemp | Have you felt happiest about your shape when your stomach has been empty |
| aubthinwomen | Felt that it is not fair that other women are thinner than you |
| aubfleshdimply | Worried about your flesh being dimply |
| aucnostopeat | I know I won't be able to stop eating once I start |
| aucuncontroleat | I often lose control and eat too much |
| aucwaytoeat | Make me think of ways to get what I want to eat |
| aucfoodonmind | I have food on my mind all the time |
| aucpreoccupied | Myself preoccupied with food |
| aucmakeplan | I find myself making plans to eat |
| aucboredangsad | When I feel bored, angry, or sad |
| aucnowillpower | No willpower to resist my food craving |
| auctroubstop | Once I start eating, I have trouble stopping |
| auccannotstop | I can't stop thinking about eating no matter how hard I try |
| auclosecontrol | If I give in to a food craving, all control is lost |
| auckeepthink | Keep on thinking about eating until I actually eat the food |
| aucthought | Thoughts of eating it consume me |
| aucemotion | My emotions often make me want to eat |
| auctempt | Hard for me to resist the temptation to eat appetizing foods that are in my reach |
| auesizzsteak | Smell a sizzling steak or see a juicy piece of meat, find it very difficult to keep from eating |
| auesocial | Eat too much at social occasions |
| aueeat3aday | I am usually so hungry that I eat more than three times a day |
| auequotacalo | Eaten my quota of calories, I am usually good about not eating anymore |
| auedieting | I just get too hungry |
| auesmhelping | Take small helpings as a means of controlling my weight |
| auetastegood | Keep on eating even when I am no longer hungry |
| aueexpert | Wish that while I am eating, an expert would tell me that I have had enough |
| aueanxious | I feel anxious, I find myself eating |
| auelifeshort | Life is too short to worry about dieting |
| auewtupdown | My weight goes up and down, have gone on reducing diets more than once |
| aueeatsomething | Feel so hungry that I just have to eat something |
| aueovereat | With someone who is overeating, I usually overeat too |
| auecalories | Good idea of the number of calories in common food |
| auenostop | When I start eating, I just can't seem to stop |
| aueleavefood | Not difficult for me to leave something on my plate |
| aueusedto | Get hungry because I have gotten used to eating then |
| auedieteatless | While on a diet, I consciously eat less for a period of time to make up for it |
| auesomeoneeat | Being with someone who is eating often makes me hungry |
| aueblue | When I feel blue, I often overeat |
| aueeattoomuch | I enjoy eating too much to spoil it by counting calories |
| auedelicacy | When I see a real delicacy, I often get so hungry |
| auelimitamt | I often stop eating when I am not really full |
| auebottomless | I get so hungry that my stomach often seems like a bottomless pit |
| auewtnochange | My weight has hardly changed at all |
| auefinishfood | I am always hungry so it is hard for me to stop eating |
| auelonely | When I feel lonely, I console myself by eating |
| aueholdback | I consciously hold back at meals in order to not gain weight |
| auehungrynight | Get very hungry late in the evening or at night |
| aueeatanything | I eat anything I want, anytime I want |
| auenothinking | Without even thinking about it, I take a long time to eat |
| auecountcalor | I count calories as a conscious means of controlling my weight |
| auenoteatsome | I do not eat some foods because they make me fat |
| aueeatanytime | I am always hungry enough to eat at any time |
| auefigure | I pay a great deal of attention to changes in my figure |
| auesplurge | I eat a food that is not allowed, I often then splurge |
| auecontrolwt | Dieting in a conscious effort to control your weight |
| auewtfluctuate | A weight fluctuation of 5 pounds affect the way you live your life |
| auefreqhung | How often do you feel hungry |
| aueguilt | Feelings of guilt about overeating help you control your food intake |
| aueeathalfway | How difficult would it be for you to stop eating halfway through dinner |
| aueconscious | How conscious are you of what you are eating |
| auestockingup | How frequently do you avoid 'stocking up' on tempting foods |
| aueshoplowcal | How likely are you to shop for low calorie foods |
| auesensibly | Do you eat sensibly in front of others and splurge alone |
| aueeatslow | Consciously eat slowly in order to cut down on how much you eat |
| aueskipdessert | How frequently do you skip dessert because you are no longer hungry |
| aueeatless | How likely are you to consciously eat less than you want |
| aueeatbinge | Do you go on eating binges though you are not hungry |
| auerestrainteating | On a scale of 0 to 5, where 0 means no restraint in eating and 5 means total restraint, what number would you give yourself |
| aueeatbehav | What extent does this statement describe eating behavior |
| aurmeetneeds | How well does your partner meet your needs |
| aursatisrelat | How satisfied are you with your relationship |
| aurgdrelat | How good is your relationship compared to most |
| aurwishno | How often do you wish you hadn't gotten into this relationship |
| aurmetexpect | To what extent has your relationship meet your original expectations |
| aurlovepartner | How much do you love your partner |
| aurproblems | How many problems are there in your relationship |
| aufnumbadul | How many adults live with you |
| aufrelationship1 | What are your relationships with these adults |
| aufrealtionship2 | What are your relationships with these adults |
| aufrelationship3 | What are your relationships with these adults |
| aufnumbchi | How many children live with you |
| aufageinyear1 | Age of these children |
| aufchilrelat1 | What are your relationships with these children |
| aufageinyear2 | Age of these children |
| aufchilrelat2 | What are your relationships with these children |
| aufageinyear3 | Age of these children |
| aufchilrelat3 | What are your relationships with these children |
| aufaginyear4 | <none> |
| aufchilrelat4 | <none> |
| aufaginyear5 | <none> |
| aufchilrelat5 | <none> |
| aufaginyear6 | <none> |
| aufchilrelat6 | <none> |
| aufaginyear7 | <none> |
| aufchilrelat7 | <none> |
| aufaginyear8 | <none> |
| aufchilrelat8 | <none> |
| aupacton | We usually act on our decisions regarding problems |
| aupdiscuss | After solve a problem, we usually discuss whether it worked or not |
| aupemotupset | We resolve most emotional upsets that come up |
| aupconfront | We confront problems involving feelings |
| aupthinkdiff | We try to think of different ways to solve problems |
| autupset | When someone is upset, the others know why |
| autcannottell | You can't tell how a person is feeling from what they are saying |
| authinting | People come right out and say things instead of hinting at them |
| autfrank | We are frank with each other |
| autangry | We don't talk to each other when we are angry |
| auttellthem | When we don't like what someone has done, we tell them |
| augmisunderstand | We misunderstand each other |
| augcrisis | In times of crisis, we can turn to each other for support |
| augsadness | We cannot talk to each other about the saddness |
| augaccepted | Individuals are accepted for what they are |
| augfear | We avoid discussing our fears and concerns |
| augexprefel | We can express feelings to each other |
| augbadfeeling | There are lots of bad feelings in the family |
| augacceptwhat | We feel accepted for what we are |
| augproblem | Making decisions is a problem for our family |
| augsolve | We are able to make decisions about how to solve problems |
| augnogetalong | We don't get along well together |
| augconfide | We confide in each other |
| acheightinches | How tall are you without shoes |
| acwt | How much did you weigh right before becoming pregnant |

| **Variable Values** | | |
| --- | --- | --- |
| Value | | Label |
| aufirstap | 1.00 | No |
|  | 2.00 | Yes |
| auhispanic | 1.00 | No |
|  | 2.00 | Yes |
| aurace | 1.00 | Black or African American |
|  | 2.00 | White |
|  | 3.00 | Asian American or Asian |
|  | 4.00 | Native Hawaiian or other Pacific Islander |
|  | 5.00 | American Indian or Alaska Native |
| ausmoke | 1.00 | Never smoked |
|  | 2.00 | Smoked, but quit |
|  | 3.00 | Smoker |
| auedu | 1.00 | 8th grade or less |
|  | 2.00 | Some high school |
|  | 3.00 | High school graduate |
|  | 4.00 | Some college or technical school |
|  | 5.00 | College graduate or higher |
| auemploy1 | 1.00 | Full time |
|  | 2.00 | Part time |
|  | 3.00 | Unemployed |
|  | 4.00 | Homemaker |
|  | 5.00 | Self-employed |
|  | 6.00 | Student |
|  | 7.00 | Other |
| ausswamped | 1.00 | Not at all |
|  | 2.00 | rarely |
|  | 3.00 | sometimes |
|  | 4.00 | often |
|  | 5.00 | A lot |
| ausnotime | 1.00 | Not at all |
|  | 2.00 | rarely |
|  | 3.00 | sometimes |
|  | 4.00 | often |
|  | 5.00 | A lot |
| ausrush | 1.00 | Not at all |
|  | 2.00 | rarely |
|  | 3.00 | sometimes |
|  | 4.00 | often |
|  | 5.00 | A lot |
| auspillingup | 1.00 | Not at all |
|  | 2.00 | rarely |
|  | 3.00 | sometimes |
|  | 4.00 | often |
|  | 5.00 | A lot |
| ausheavyload | 1.00 | Not at all |
|  | 2.00 | rarely |
|  | 3.00 | sometimes |
|  | 4.00 | often |
|  | 5.00 | A lot |
| ausinadequate | 1.00 | Not at all |
|  | 2.00 | rarely |
|  | 3.00 | sometimes |
|  | 4.00 | often |
|  | 5.00 | A lot |
| ausodds | 1.00 | Not at all |
|  | 2.00 | rarely |
|  | 3.00 | sometimes |
|  | 4.00 | often |
|  | 5.00 | A lot |
| ausnothing | 1.00 | Not at all |
|  | 2.00 | rarely |
|  | 3.00 | sometimes |
|  | 4.00 | often |
|  | 5.00 | A lot |
| ausnoescape | 1.00 | Not at all |
|  | 2.00 | rarely |
|  | 3.00 | sometimes |
|  | 4.00 | often |
|  | 5.00 | A lot |
| ausgivingup | 1.00 | Not at all |
|  | 2.00 | rarely |
|  | 3.00 | sometimes |
|  | 4.00 | often |
|  | 5.00 | A lot |
| auanervous | .00 | Not at all sure |
|  | 1.00 | Several days |
|  | 2.00 | Over half the days |
|  | 3.00 | Nearly every day |
| auastopworry | .00 | Not at all sure |
|  | 1.00 | Several days |
|  | 2.00 | Over half the days |
|  | 3.00 | Nearly every day |
| auaworrymuch | .00 | Not at all sure |
|  | 1.00 | Several days |
|  | 2.00 | Over half the days |
|  | 3.00 | Nearly every day |
| auatrourelax | .00 | Not at all sure |
|  | 1.00 | Several days |
|  | 2.00 | Over half the days |
|  | 3.00 | Nearly every day |
| auarestless | .00 | Not at all sure |
|  | 1.00 | Several days |
|  | 2.00 | Over half the days |
|  | 3.00 | Nearly every day |
| auaannoyed | .00 | Not at all sure |
|  | 1.00 | Several days |
|  | 2.00 | Over half the days |
|  | 3.00 | Nearly every day |
| auaafraid | .00 | Not at all sure |
|  | 1.00 | Several days |
|  | 2.00 | Over half the days |
|  | 3.00 | Nearly every day |
| auadifficult | .00 | Not at all sure |
|  | 1.00 | Several days |
|  | 2.00 | Over half the days |
|  | 3.00 | Nearly every day |
| audlaugh | .00 | Not at all |
|  | 1.00 | Definitely not so much now |
|  | 2.00 | Not quite so much now |
|  | 3.00 | As much as I always could |
| audenjoyment | .00 | Hardly at all |
|  | 1.00 | Definitely less than I used to |
|  | 2.00 | Rather less than I used to |
|  | 3.00 | As much as I ever did |
| audblamedme | .00 | No, never |
|  | 1.00 | Not very often |
|  | 2.00 | Yes, some of the time |
|  | 3.00 | Yes, most of the time |
| audanxious | .00 | Yes, very often |
|  | 1.00 | Yes, sometimes |
|  | 2.00 | Hardly ever |
|  | 3.00 | No, not at all |
| audscared | .00 | No, not at all |
|  | 1.00 | No, not much |
|  | 2.00 | Yes, sometimes |
|  | 3.00 | Yes, quite a lot |
| audontopofme | .00 | No, I have been coping as well as ever |
|  | 1.00 | No, most of the time I have coped quite well |
|  | 2.00 | Yes, sometimes I haven't been coping as well as usual |
|  | 3.00 | Yes, most of the time I haven't been able to cope at all |
| audunhappys | .00 | No, not at all |
|  | 1.00 | Not very often |
|  | 2.00 | Yes, sometimes |
|  | 3.00 | Yes, most of the time |
| audsad | .00 | No, not at all |
|  | 1.00 | Not very often |
|  | 2.00 | Yes, quite often |
|  | 3.00 | Yes, most of the time |
| audunhappyc | .00 | No, never |
|  | 1.00 | Only occasionally |
|  | 2.00 | Yes, quite often |
|  | 3.00 | Yes, most of the time |
| audharmingme | .00 | Never |
|  | 1.00 | Hardly ever |
|  | 2.00 | Sometimes |
|  | 3.00 | Yes, quite often |
| aubfleshfirm | 1.00 | Never |
|  | 2.00 | Rarely |
|  | 3.00 | Sometimes |
|  | 4.00 | Often |
|  | 5.00 | Very Often |
|  | 6.00 | Always |
| aubsmamtfood | 1.00 | Never |
|  | 2.00 | Rarely |
|  | 3.00 | Sometimes |
|  | 4.00 | Often |
|  | 5.00 | Very Often |
|  | 6.00 | Always |
| aubavoidclo | 1.00 | Never |
|  | 2.00 | Rarely |
|  | 3.00 | Sometimes |
|  | 4.00 | Often |
|  | 5.00 | Very Often |
|  | 6.00 | Always |
| aubashame | 1.00 | Never |
|  | 2.00 | Rarely |
|  | 3.00 | Sometimes |
|  | 4.00 | Often |
|  | 5.00 | Very Often |
|  | 6.00 | Always |
| aubworryshape | 1.00 | Never |
|  | 2.00 | Rarely |
|  | 3.00 | Sometimes |
|  | 4.00 | Often |
|  | 5.00 | Very Often |
|  | 6.00 | Always |
| aubstomachemp | 1.00 | Never |
|  | 2.00 | Rarely |
|  | 3.00 | Sometimes |
|  | 4.00 | Often |
|  | 5.00 | Very Often |
|  | 6.00 | Always |
| aubthinwomen | 1.00 | Never |
|  | 2.00 | Rarely |
|  | 3.00 | Sometimes |
|  | 4.00 | Often |
|  | 5.00 | Very Often |
|  | 6.00 | Always |
| aubfleshdimply | 1.00 | Never |
|  | 2.00 | Rarely |
|  | 3.00 | Sometimes |
|  | 4.00 | Often |
|  | 5.00 | Very Often |
|  | 6.00 | Always |
| aucnostopeat | 1.00 | Strongly Disagree |
|  | 2.00 | Disagree |
|  | 3.00 | Neutral |
|  | 4.00 | Agree |
|  | 5.00 | Strongly Agree |
| aucuncontroleat | 1.00 | Strongly Disagree |
|  | 2.00 | Disagree |
|  | 3.00 | Neutral |
|  | 4.00 | Agree |
|  | 5.00 | Strongly Agree |
| aucwaytoeat | 1.00 | Strongly Disagree |
|  | 2.00 | Disagree |
|  | 3.00 | Neutral |
|  | 4.00 | Agree |
|  | 5.00 | Strongly Agree |
| aucfoodonmind | 1.00 | Strongly Disagree |
|  | 2.00 | Disagree |
|  | 3.00 | Neutral |
|  | 4.00 | Agree |
|  | 5.00 | Strongly Agree |
| aucpreoccupied | 1.00 | Strongly Disagree |
|  | 2.00 | Disagree |
|  | 3.00 | Neutral |
|  | 4.00 | Agree |
|  | 5.00 | Strongly Agree |
| aucmakeplan | 1.00 | Strongly Disagree |
|  | 2.00 | Disagree |
|  | 3.00 | Neutral |
|  | 4.00 | Agree |
|  | 5.00 | Strongly Agree |
| aucboredangsad | 1.00 | Strongly Disagree |
|  | 2.00 | Disagree |
|  | 3.00 | Neutral |
|  | 4.00 | Agree |
|  | 5.00 | Strongly Agree |
| aucnowillpower | 1.00 | Strongly Disagree |
|  | 2.00 | Disagree |
|  | 3.00 | Neutral |
|  | 4.00 | Agree |
|  | 5.00 | Strongly Agree |
| auctroubstop | 1.00 | Strongly Disagree |
|  | 2.00 | Disagree |
|  | 3.00 | Neutral |
|  | 4.00 | Agree |
|  | 5.00 | Strongly Agree |
| auccannotstop | 1.00 | Strongly Disagree |
|  | 2.00 | Disagree |
|  | 3.00 | Neutral |
|  | 4.00 | Agree |
|  | 5.00 | Strongly Agree |
| auclosecontrol | 1.00 | Strongly Disagree |
|  | 2.00 | Disagree |
|  | 3.00 | Neutral |
|  | 4.00 | Agree |
|  | 5.00 | Strongly Agree |
| auckeepthink | 1.00 | Strongly Disagree |
|  | 2.00 | Disagree |
|  | 3.00 | Neutral |
|  | 4.00 | Agree |
|  | 5.00 | Strongly Agree |
| aucthought | 1.00 | Strongly Disagree |
|  | 2.00 | Disagree |
|  | 3.00 | Neutral |
|  | 4.00 | Agree |
|  | 5.00 | Strongly Agree |
| aucemotion | 1.00 | Strongly Disagree |
|  | 2.00 | Disagree |
|  | 3.00 | Neutral |
|  | 4.00 | Agree |
|  | 5.00 | Strongly Agree |
| auctempt | 1.00 | Strongly Disagree |
|  | 2.00 | Disagree |
|  | 3.00 | Neutral |
|  | 4.00 | Agree |
|  | 5.00 | Strongly Agree |
| auesizzsteak | .00 | False |
|  | 1.00 | True |
| auesocial | .00 | False |
|  | 1.00 | True |
| aueeat3aday | .00 | False |
|  | 1.00 | True |
| auequotacalo | .00 | False |
|  | 1.00 | True |
| auedieting | .00 | False |
|  | 1.00 | True |
| auesmhelping | .00 | False |
|  | 1.00 | True |
| auetastegood | .00 | False |
|  | 1.00 | True |
| aueexpert | .00 | False |
|  | 1.00 | True |
| aueanxious | .00 | False |
|  | 1.00 | True |
| auelifeshort | .00 | False |
|  | 1.00 | True |
| auewtupdown | .00 | False |
|  | 1.00 | True |
| aueeatsomething | .00 | False |
|  | 1.00 | True |
| aueovereat | .00 | False |
|  | 1.00 | True |
| auecalories | .00 | False |
|  | 1.00 | True |
| auenostop | .00 | False |
|  | 1.00 | True |
| aueleavefood | .00 | False |
|  | 1.00 | True |
| aueusedto | .00 | False |
|  | 1.00 | True |
| auedieteatless | .00 | False |
|  | 1.00 | True |
| auesomeoneeat | .00 | False |
|  | 1.00 | True |
| aueblue | .00 | False |
|  | 1.00 | True |
| aueeattoomuch | .00 | False |
|  | 1.00 | True |
| auedelicacy | .00 | False |
|  | 1.00 | True |
| auelimitamt | .00 | False |
|  | 1.00 | True |
| auebottomless | .00 | False |
|  | 1.00 | True |
| auewtnochange | .00 | False |
|  | 1.00 | True |
| auefinishfood | .00 | False |
|  | 1.00 | True |
| auelonely | .00 | False |
|  | 1.00 | True |
| aueholdback | .00 | False |
|  | 1.00 | True |
| auehungrynight | .00 | False |
|  | 1.00 | True |
| aueeatanything | .00 | False |
|  | 1.00 | True |
| auenothinking | .00 | False |
|  | 1.00 | True |
| auecountcalor | .00 | False |
|  | 1.00 | True |
| auenoteatsome | .00 | False |
|  | 1.00 | True |
| aueeatanytime | .00 | False |
|  | 1.00 | True |
| auefigure | .00 | False |
|  | 1.00 | True |
| auesplurge | .00 | False |
|  | 1.00 | True |
| auecontrolwt | 1.00 | Rarely |
|  | 2.00 | Sometimes |
|  | 3.00 | Usually |
|  | 4.00 | Always |
| auewtfluctuate | 1.00 | Not at all |
|  | 2.00 | Slightly |
|  | 3.00 | Moderately |
|  | 4.00 | Very Much |
| auefreqhung | 1.00 | Only at mealtimes |
|  | 2.00 | Sometimes between meals |
|  | 3.00 | Often between meals |
|  | 4.00 | Almost always |
| aueguilt | 1.00 | Never |
|  | 2.00 | Rarely |
|  | 3.00 | Often |
|  | 4.00 | Always |
| aueeathalfway | 1.00 | Easy |
|  | 2.00 | Slightly difficult |
|  | 3.00 | Moderately difficult |
|  | 4.00 | Very difficult |
| aueconscious | 1.00 | Not at all |
|  | 2.00 | Slightly |
|  | 3.00 | Moderately |
|  | 4.00 | Extremely |
| auestockingup | 1.00 | Almost never |
|  | 2.00 | Seldom |
|  | 3.00 | Usually |
|  | 4.00 | Almost always |
| aueshoplowcal | 1.00 | Unlikely |
|  | 2.00 | Slightly unlikely |
|  | 3.00 | Moderately likely |
|  | 4.00 | Very likely |
| auesensibly | 1.00 | Never |
|  | 2.00 | Rarely |
|  | 3.00 | Often |
|  | 4.00 | Always |
| aueeatslow | 1.00 | Never |
|  | 2.00 | Slightly unlikely |
|  | 3.00 | Moderately likely |
|  | 4.00 | Very likely |
| aueskipdessert | 1.00 | Almost never |
|  | 2.00 | Seldom |
|  | 3.00 | At least once a week |
|  | 4.00 | Almost every day |
| aueeatless | 1.00 | Unlikely |
|  | 2.00 | Slightly unlikely |
|  | 3.00 | Moderately likely |
|  | 4.00 | Very likely |
| aueeatbinge | 1.00 | Never |
|  | 2.00 | Rarely |
|  | 3.00 | Sometimes |
|  | 4.00 | At least once a week |
| auerestrainteating | .00 | Eat whatever you want, whenever you want it |
|  | 1.00 | Usually eat whatever you want, whenever you want it |
|  | 2.00 | Often eat whatever you want, whenever you want it |
|  | 3.00 | Often limit food intake, but often 'give in' |
|  | 4.00 | Usually limit food intake, rarely 'give in' |
|  | 5.00 | Constantly limiting food intake, never 'give in' |
| aueeatbehav | 1.00 | Not like me |
|  | 2.00 | Little like me |
|  | 3.00 | Pretty good description of me |
|  | 4.00 | Describes me perfectly |
| aurmeetneeds | .00 | Not applicable |
|  | 1.00 | Poorly |
|  | 2.00 | fair |
|  | 3.00 | Average |
|  | 4.00 | well |
|  | 5.00 | Extremely well |
| aursatisrelat | .00 | Not applicable |
|  | 1.00 | Poorly |
|  | 2.00 | fair |
|  | 3.00 | Average |
|  | 4.00 | well |
|  | 5.00 | Extremely well |
| aurgdrelat | .00 | Not applicable |
|  | 1.00 | Poorly |
|  | 2.00 | fair |
|  | 3.00 | Average |
|  | 4.00 | well |
|  | 5.00 | Extremely well |
| aurwishno | .00 | Not applicable |
|  | 1.00 | Poorly |
|  | 2.00 | fair |
|  | 3.00 | Average |
|  | 4.00 | well |
|  | 5.00 | Extremely well |
| aurmetexpect | .00 | Not applicable |
|  | 1.00 | Poorly |
|  | 2.00 | fair |
|  | 3.00 | Average |
|  | 4.00 | well |
|  | 5.00 | Extremely well |
| aurlovepartner | .00 | Not applicable |
|  | 1.00 | Poorly |
|  | 2.00 | fair |
|  | 3.00 | Average |
|  | 4.00 | well |
|  | 5.00 | Extremely well |
| aurproblems | .00 | Not applicable |
|  | 1.00 | Poorly |
|  | 2.00 | fair |
|  | 3.00 | Average |
|  | 4.00 | well |
|  | 5.00 | Extremely well |
| aufrelationship1 | 1.00 | Mother |
|  | 2.00 | Husband/partner/significant other |
|  | 3.00 | Father |
|  | 4.00 | Grandmother |
|  | 5.00 | Sister |
|  | 6.00 | Brother |
|  | 7.00 | Grandfather |
|  | 8.00 | Friend |
|  | 9.00 | Cousin |
|  | 10.00 | Niece |
|  | 11.00 | Nephew |
|  | 12.00 | Other |
| aufrealtionship2 | 1.00 | Mother |
|  | 2.00 | Husband/partner/significant other |
|  | 3.00 | Father |
|  | 4.00 | Grandmother |
|  | 5.00 | Sister |
|  | 6.00 | Brother |
|  | 7.00 | Grandfather |
|  | 8.00 | Friend |
|  | 9.00 | Cousin |
|  | 10.00 | Niece |
|  | 11.00 | Nephew |
|  | 12.00 | Other |
| aufrelationship3 | 1.00 | Mother |
|  | 2.00 | Husband/partner/significant other |
|  | 3.00 | Father |
|  | 4.00 | Grandmother |
|  | 5.00 | Sister |
|  | 6.00 | Brother |
|  | 7.00 | Grandfather |
|  | 8.00 | Friend |
|  | 9.00 | Cousin |
|  | 10.00 | Niece |
|  | 11.00 | Nephew |
|  | 12.00 | Other |
| aufchilrelat1 | 1.00 | Son |
|  | 2.00 | Daughter |
|  | 3.00 | Cousin |
|  | 4.00 | Niece |
|  | 5.00 | Nephew |
|  | 6.00 | Step son |
|  | 7.00 | Step daughter |
|  | 8.00 | Sister |
|  | 9.00 | Brother |
|  | 10.00 | Other |
| aufchilrelat2 | 1.00 | Son |
|  | 2.00 | Daughter |
|  | 3.00 | Cousin |
|  | 4.00 | Niece |
|  | 5.00 | Nephew |
|  | 6.00 | Step son |
|  | 7.00 | Step daughter |
|  | 8.00 | Sister |
|  | 9.00 | Brother |
|  | 10.00 | Other |
| aufchilrelat3 | 1.0000 | Son |
|  | 2.0000 | Daughter |
|  | 3.0000 | Cousin |
|  | 4.0000 | Niece |
|  | 5.0000 | Nephew |
|  | 6.0000 | Step son |
|  | 7.0000 | Step daughter |
|  | 8.0000 | Sister |
|  | 9.0000 | Brother |
|  | 10.0000 | Other |
| aufchilrelat4 | 1.00 | Son |
|  | 2.00 | Daughter |
|  | 3.00 | Cousin |
|  | 4.00 | Niece |
|  | 5.00 | Nephew |
|  | 6.00 | Step son |
|  | 7.00 | Step daughter |
|  | 8.00 | Sister |
|  | 9.00 | Brother |
|  | 10.00 | Other |
| aufchilrelat5 | 1.00 | Son |
|  | 2.00 | Daughter |
|  | 3.00 | Cousin |
|  | 4.00 | Niece |
|  | 5.00 | Nephew |
|  | 6.00 | Step son |
|  | 7.00 | Step daughter |
|  | 8.00 | Sister |
|  | 9.00 | Brother |
|  | 10.00 | Other |
| aufchilrelat6 | 1.00 | Son |
|  | 2.00 | Daughter |
|  | 3.00 | Cousin |
|  | 4.00 | Niece |
|  | 5.00 | Nephew |
|  | 6.00 | Step son |
|  | 7.00 | Step daughter |
|  | 8.00 | Sister |
|  | 9.00 | Brother |
|  | 10.00 | Other |
| aufchilrelat7 | 1.00 | Son |
|  | 2.00 | Daughter |
|  | 3.00 | Cousin |
|  | 4.00 | Niece |
|  | 5.00 | Nephew |
|  | 6.00 | Step son |
|  | 7.00 | Step daughter |
|  | 8.00 | Sister |
|  | 9.00 | Brother |
|  | 10.00 | Other |
| aufchilrelat8 | 1.00 | Son |
|  | 2.00 | Daughter |
|  | 3.00 | Cousin |
|  | 4.00 | Niece |
|  | 5.00 | Nephew |
|  | 6.00 | Step son |
|  | 7.00 | Step daughter |
|  | 8.00 | Sister |
|  | 9.00 | Brother |
|  | 10.00 | Other |
| aupacton | 1.00 | Strongly Disagree |
|  | 2.00 | Disagree |
|  | 3.00 | Agree |
|  | 4.00 | Strongly agree |
| aupdiscuss | 1.00 | Strongly Disagree |
|  | 2.00 | Disagree |
|  | 3.00 | Agree |
|  | 4.00 | Strongly agree |
| aupemotupset | 1.00 | Strongly Disagree |
|  | 2.00 | Disagree |
|  | 3.00 | Agree |
|  | 4.00 | Strongly agree |
| aupconfront | 1.00 | Strongly Disagree |
|  | 2.00 | Disagree |
|  | 3.00 | Agree |
|  | 4.00 | Strongly agree |
| aupthinkdiff | 1.00 | Strongly Disagree |
|  | 2.00 | Disagree |
|  | 3.00 | Agree |
|  | 4.00 | Strongly agree |
| autupset | 1.00 | Strongly Disagree |
|  | 2.00 | Disagree |
|  | 3.00 | Agree |
|  | 4.00 | Strongly agree |
| autcannottell | 1.00 | Strongly Disagree |
|  | 2.00 | Disagree |
|  | 3.00 | Agree |
|  | 4.00 | Strongly agree |
| authinting | 1.00 | Strongly Disagree |
|  | 2.00 | Disagree |
|  | 3.00 | Agree |
|  | 4.00 | Strongly agree |
| autfrank | 1.00 | Strongly Disagree |
|  | 2.00 | Disagree |
|  | 3.00 | Agree |
|  | 4.00 | Strongly agree |
| autangry | 1.00 | Strongly Disagree |
|  | 2.00 | Disagree |
|  | 3.00 | Agree |
|  | 4.00 | Strongly agree |
| auttellthem | 1.00 | Strongly Disagree |
|  | 2.00 | Disagree |
|  | 3.00 | Agree |
|  | 4.00 | Strongly agree |
| augmisunderstand | 1.00 | Strongly Disagree |
|  | 2.00 | Disagree |
|  | 3.00 | Agree |
|  | 4.00 | Strongly agree |
| augcrisis | 1.00 | Strongly Disagree |
|  | 2.00 | Disagree |
|  | 3.00 | Agree |
|  | 4.00 | Strongly agree |
| augsadness | 1.00 | Strongly Disagree |
|  | 2.00 | Disagree |
|  | 3.00 | Agree |
|  | 4.00 | Strongly agree |
| augaccepted | 1.00 | Strongly Disagree |
|  | 2.00 | Disagree |
|  | 3.00 | Agree |
|  | 4.00 | Strongly agree |
| augfear | 1.00 | Strongly Disagree |
|  | 2.00 | Disagree |
|  | 3.00 | Agree |
|  | 4.00 | Strongly agree |
| augexprefel | 1.00 | Strongly Disagree |
|  | 2.00 | Disagree |
|  | 3.00 | Agree |
|  | 4.00 | Strongly agree |
| augbadfeeling | 1.00 | Strongly Disagree |
|  | 2.00 | Disagree |
|  | 3.00 | Agree |
|  | 4.00 | Strongly agree |
| augacceptwhat | 1.00 | Strongly Disagree |
|  | 2.00 | Disagree |
|  | 3.00 | Agree |
|  | 4.00 | Strongly agree |
| augproblem | 1.00 | Strongly Disagree |
|  | 2.00 | Disagree |
|  | 3.00 | Agree |
|  | 4.00 | Strongly agree |
| augsolve | 1.00 | Strongly Disagree |
|  | 2.00 | Disagree |
|  | 3.00 | Agree |
|  | 4.00 | Strongly agree |
| augnogetalong | 1.00 | Strongly Disagree |
|  | 2.00 | Disagree |
|  | 3.00 | Agree |
|  | 4.00 | Strongly agree |
| augconfide | 1.00 | Strongly Disagree |
|  | 2.00 | Disagree |
|  | 3.00 | Agree |
|  | 4.00 | Strongly agree |
